# Association between plasma concentrations of linoleic acid-derived oxylipins and the perceived pain scores in an exploratory study in women with chronic neck pain

**DOI:** 10.1186/s12891-016-0951-9

**Published:** 2016-02-25

**Authors:** Fredrik Hellström, Sandra Gouveia-Figueira, Malin L. Nording, Martin Björklund, Christopher J. Fowler

**Affiliations:** Department of Occupational and Public Health Sciences, Centre for Musculoskeletal Research, University of Gävle, SE 907 13 Umeå, Sweden; Department of Chemistry, Umeå University, SE-901 87 Umeå, Sweden; Department of Pharmacology and Clinical Neuroscience, Umeå University, SE-901 87 Umeå, Sweden; Department of Community Medicine and Rehabilitation, Umeå University, Umeå, Sweden

**Keywords:** Musculoskeletal disorders, chronic neck pain, chronic widespread pain, endocannabinoids, *N*-acylethanolamines, oxylipins, 9-hydroxy-10*E*,12*Z*-octadecadienoic acid, 13-hydroxy-9*Z*,11*E*-octadecadienoic acid

## Abstract

**Background:**

Chronic musculoskeletal pain may be associated with changes in the balance of algogenic and anti-nociceptive compounds, and such changes may be visible in plasma samples. We have undertaken an exploratory study to measure the levels of endocannabinoids, related *N*-acylethanolamines and oxylipins (primarily those derived from linoleic acid) in plasma samples from women with chronic neck pain (NP) and chronic widespread pain (CWP), and to investigate whether the observed levels are associated with the pain experienced by these women.

**Methods:**

Blood samples from 35 women with NP, 15 with CWP and 27 age-matched controls were analysed for the lipids using ultra performance liquid chromatography coupled to tandem mass spectrometry. Current pain (“NRS_day_”) and the average pain during the last week (“NRS_week_”) were rated by the participants using a numerical rating scale.

**Results:**

There were no significant differences in the plasma concentrations of the fifteen lipids investigated between the women with pain and the controls. However, significant correlations were seen for the NP group between the NRS_day_ scores and the plasma concentrations of the linoleic acid derivatives 9- and 13-hydroxy-octadecadienoic acid (Spearman’s rho values 0.51 [*P* = 0.0016]) and 0.53 [*P* = 0.0011], respectively).

**Conclusions:**

The data obtained in this exploratory study indicate that although no group differences are seen in plasma lipid concentrations, there is an association between the NRS_day_ scores and the 9- and 13-hydroxy-octadecadienoic acid levels. Whether or not the association reflects a causality (i.e. that the circulating lipids contribute to the perceived pain of the pain participants), requires further investigation.

## Background

Chronic pain is a very common condition with a large cost to society both in economic and personal terms. Chronic pain, which most often presents as musculoskeletal pain, can be either localised to defined structures or areas or have a more widespread occurrence. Localised pain may be both specific and nonspecific, i.e. the presence of specific diagnosis or not. Pain localised to the neck (NP) was the fourth ranked disorder with respect to years lived with disability in the USA during the period 1990–2010 [1]. Chronic widespread pain (CWP), defined by the American College of Rheumatology as pain present on both sides of the body as well as both above and below the waist [2, 3] is also very common, with a prevalence in the range 4.8–7.4 % of the population [4]. The pain in CWP and NP has both central and peripheral components, and at the level of the muscle, higher levels of algogenic compounds like glutamate and lactate have been reported in these conditions [5]. Interestingly, the raised interstitial concentrations of glutamate and lactate seen in muscle dialysates from CWP patients were also seen in plasma from the same individuals [5]. In another microdialysis study investigating controls and individuals with trapezius myalgia, a positive correlation was seen between baseline pain and the interstitial levels of serotonin, an important pain-signalling molecule [6].

Endogenous pain modulation is not restricted to glutamate, lactate and serotonin alone, and there are a number of lipids, including *N*-acylethanolamines (NAEs), endocannabinoids and oxylipins derived from linoleic acid that affect pain perception. With respect to the NAEs, the most well-studied is arachidonoylethanolamide (AEA, anandamide), which produces its actions in the body primarily via effects upon cannabinoid (CB) receptors, although it can also act upon transient receptor potential vanilloid 1 (TRPV1) receptors, particularly under conditions of inflammation [7]. A related compound, 2-arachidonoylglycerol (2-AG) is also an endocannabinoid, and compounds inhibiting the hydrolysis of either AEA or 2-AG are active in a wide variety of animal models of pain [8, 9]. The NAE palmitoylethanolamide (PEA) is not an endocannabinoid, but produces analgesia purportedly by activation of peroxisome proliferator-activated receptor α [10]. Oleoylethanolamide (OEA) also affects pain, albeit by a mechanism independent of peroxisome proliferator-activated receptor α [11]. In addition to its analgesic effects, PEA is anti-inflammatory, a property shared by stearoylethanolamide (SEA) [12]. Other members of the NAE family, such as linoleoylethanolamide (LEA), are less well investigated.

Relatively little is known about NAE/2-AG levels in human musculoskeletal pain. However, in trapezius muscle microdialysates, levels of PEA and SEA are increased in chronic neck/shoulder pain, but not in CWP [13]. It is not known whether changes in the levels of these NAEs are seen in the plasma of individuals with CWP and/or chronic neck pain (NP). A large increase in plasma AEA, however, has been reported in patients with fibromyalgia [14].

Oxylipins derived from linoleic acid are another important class of biologically active lipids. In plasma, among the most prevalent are 9-HODE (9-hydroxy-10*E*,12*Z*-octadecadienoic acid) and 13-HODE (13-hydroxy-9*Z*,11*E*-octadecadienoic acid). To our knowledge, it is not known whether plasma/serum levels of the HODEs or related lipids are changed in human musculoskeletal pain, but they are increased in other pain conditions, such as Achilles tendinopathy [15]. Given the ability of these compounds to activate TRPV1 receptors involved in pain transmission [16, 17], it is possible that the increased levels of these lipids may contribute to the pain found in these disorders.

From the above, it can be argued that chronic musculoskeletal pain may be associated with changes in the balance of algogenic and anti-nociceptive compounds, and that such changes may be visible in plasma samples. In consequence, given the lack of knowledge in this area, we have undertaken an exploratory study to measure the levels of AEA, 2-AG, related NAEs and linoleic acid-derived oxylipins in plasma samples from healthy women, women with CWP and NP, and to investigate whether the observed levels are associated with the pain experienced by these individuals.

## Methods

### Subjects

Female participants of age range 20–65 years were consecutively recruited during the period June 2011–March 2012. Twenty-seven healthy controls and 36 subjects with NP (of whom data from 35 are reported here) were recruited from a randomized controlled trial (Current Controlled Trials registration ISRCTN49348025; the trial comprised 120 subjects with NP and 40 healthy controls) [18]. Seventeen subjects with CWP (of whom data from 15 are reported here) were recruited via contact with the local patient organisation and/or advertising in local newspapers. Ethical approval for this project was granted by the ethical committee of Uppsala University (registration number 2011/081). All subjects participated voluntarily after informed written consent. For the NP group, inclusion criteria were more than 6 weeks of non-specific neck-shoulder pain (indicated as dominant pain area in a pain drawing), more than “no disability” but less than “complete disability” according to the Neck Disability Index (NDI), and self-reported impaired productivity to work the preceding month (for details see [18]). The CWP group had obtained their diagnosis using the American College of Rheumatology criteria for fibromyalgia [2] albeit without the requirement of at least 11/18 trigger points. Subjects with rheumatoid arthritis, systemic lupus erythematosus, Bechterew’s disease, multiple sclerosis, epilepsy or Parkinson’s disease, type I-diabetes, cardiovascular disease or endocrine diseases were excluded, as were subjects who did not eat either fish or meat on a regular basis.

All participants were asked not to use any pain medications except for paracetamol preparations three days before the blood sampling and to avoid intake of caffeine, nicotine and wholegrain during the 12 h prior to blood sampling. The subjects were allowed water alone during the last two hours of this period and were also asked not to perform any shoulder or neck-straining exercises for the last two days prior to blood sampling, except for ordinary daily work and/or leisure activities. All subjects rated the current pain (“NRS_day_”) and the average pain during the last week (“NRS_week_”) on a numerical rating scale [19] (0–10, where 0 is no pain and 10 is the worst pain imaginable) at the time of blood sample collection. NRS is adjudged to have a higher validity than several other commonly used pain scores [20].

### Blood sampling procedure

Blood was sampled either during the morning (between 07:30 and 11:30) or afternoon (between 12:00 and 15:00). After arrival at the laboratory, subjects were allowed to rest for 15 min before venous blood was drawn into Li-Heparin prepared Vacutainer tubes from the bend of the arm while the subjects were in a sitting position. Blood for plasma analysis were treated according to a standardised protocol, whereby the Vacutainer tube was turned 10 times during a 30 s period at room temperature before being immediately centrifuged at 4 °C for 5 min at 2500 rpm. Supernatants were aliquoted (500 μL) into pre- labelled homopolymer tubes and frozen at −80 °C until analysis. Thus, the lipid analyses conducted here were undertaken on previously unthawed samples.

### Chemicals and reagents for lipid analyses

The following native and deuterated standards were purchased from Cayman Chemical (Ann Arbor, MI, USA): 2-AG, AEA, PEA, SEA, OEA, LEA, 9-HODE, 13-HODE, 9(10)-DiHOME (9(10)-dihydroxy-12Z-octadecenoic acid), 12(13)-DiHOME (12(13)-dihydroxy-9Z-octadecenoic acid), 13-oxo-ODE (13-oxo-9*Z*,11*E*-octadecadienoic acid), 5-HETE (5-hydroxy-6*E*,8*Z*,11*Z*,14*Z*-eicosatetraenoic acid), 8,9-DiHETrE (8,9-dihydroxy-5*Z*,11*Z*,14*Z*-eicosatrienoic acid), 12-[[(cyclohexylamino)carbonyl]amino]-dodecanoic acid (CUDA), 2-AG-d_8_, AEA-d_8_, PEA-d_4_, SEA-d_3_, OEA-d_4_, TXB_2_-d_4_, 12(13)-DiHOME-d_4_, 9(*S*)-HODE-d_4_, 20-HETE-d_4_, 5(*S*)-HETE-d_8_ and 12(13)-EpOME-d_4_. 9,10,13-TriHOME (9,10,13-trihydroxy-11-octadecenoic acid) and 9,12,13-TriHOME (9,12,13-trihydroxy-10*E*-octadecenoic acid) were obtained from Larodan (Sweden, Malmö). Butylhydroxytoluene (BHT) was obtained from the Cayman Chemical Co. All solvents and chemicals used were of HPLC grade or higher. A Milli-Q Gradient system (Millipore, Milford, MA, USA) was used to purify water.

The deuterated compounds were used as internal standards and added prior to extraction to mimic the isolation of the endogenous compounds from the plasma samples. For each native compound, a suitable internal standard was selected based on structural similarities for quantification purposes. Recovery rates of each internal standard were calculated by adding a known amount of the recovery standard CUDA in the last step before injection. The recovery rates (%, means ± SD) of the internal standards in the present study were: 2-AG-d_8_ (92.3 ± 13.3, *N* = 74), AEA-d_8_ (70.7 ± 11.3, *N* = 74), PEA-d_4_ (70.0 ± 21.8, *N* = 74), SEA-d_3_ (71.2 ± 6.2, *N* = 74), OEA-d_4_ (62.1 ± 13.7, *N* = 74), TXB_2_-d_4_ (71.7 ± 16.6, *N* = 74), 12(13)-DiHOME-d_4_ (77.2 ± 10.2, *N* = 74), 9(S)-HODE-d_4_ (67.5 ± 16.4, *N* = 74), 20-HETE-d_4_ (101.3 ± 12.2, *N* = 74), 5(S)-HETE-d_8_ (81.1 ± 14.7, *N* = 74) and 12(13)-EpOME-d_4_ (103.7 ± 13.7, *N* = 74).

### Lipid extraction and analyses

A previously reported and validated SPE protocol was used for isolation of the lipids [21]. In brief, plasma samples (400 μL) were thawed on ice and spiked with 10 μL internal standards solutions and 10 μL antioxidant (0.2 mg/mL BHT/EDTA in methanol:water (1:1)) solution before being extracted using Waters Oasis HLB cartridges (60 mg of sorbent, 30 μm particle size). Eluates were reconstituted in 100 μL of MeOH spiked with 10 μL CUDA (0.05 μg/mL) and transferred to vials before analysis using an ultra performance liquid chromatography coupled to tandem mass spectrometry (UPLC-MS/MS) method.

Lipid analyses were undertaken on an Agilent UPLC (Infinity 1290) coupled with an electrospray ionization (ESI) source to an Agilent 6490 Triple Quadrupole system equipped with the iFunnel Technology (Agilent Technologies, Santa Clara, CA, USA). Analyte separation was achieved using a Waters BEH C_18_ column (2.1 mm x 150 mm, 130 Å, 1.7 μm particle size) according to a previously validated analytical protocol [21]. Injection volumes of 10 μL were employed, and the mobile phases consisted of (A) 0.1 % acetic acid in MilliQ water and (B) acetonitrile:isopropanol (90:10). For the endocannabinoids and related NAEs, the system operated in positive mode (ESI+) with the following gradient: 0.0–2.0 min 30–45 % B, 2.0–2.5 min 45–79 % B, 2.5–11.5 min 79 % B, 11.5–12 min 79–90 % B, 12–14 min 90 % B, 14–14.5 min 90–79 % B, 14.5–15.5 min 79 % B, 15.6–19 min 30 % B. For the linoleic acid- and arachidonic acid-derived oxylipins, the negative ionisation mode was used (ESI-), with the following gradient: 0.0–3.5 min 10–35 % B, 3.5–5.5 min 40 % B, 5.5–7.0 min 42% B, 7.0–9.0 min 50 % B, 9.0–15.0 min 65 % B, 15.0–17.0 min 75 % B, 17.0–18.5 min 85 % B, 18.5–19.5 min 95 % B, 19.5–21 min 95-10 % B, 21.0–25.0 min 10 % B. The dynamic multiple reaction monitoring (dMRM) option was performed for the lipids with optimized transitions and collision energies [21]. All peaks were integrated manually using the MassHunter Workstation software, and the stable isotope dilution method was used to quantify the peaks using calibration curves of peak areas of native compounds divided by the corresponding IS peak areas. Accuracy and precision for the method is described elsewhere [21], but in general at the levels detected in the current study the precision ranged from 4.6–9.8 % (endocannabinoids and related NAEs) and 2.1–11.2 % (oxylipins) and accuracies ranged from 98–119 % (endocannabinoids and related NAEs) to 100–111 % (oxylipins).

### Statistics

Groups (both patient characteristics and outcome variables) were tested for normal distribution using the D’Agostino and Pearson normality test and differences between groups were tested using Kruskal-Wallis test, Mann Whintney *U*-test or Chi squared test, all using GraphPad Prism v. 6 for the Macintosh (GraphPad Software Inc., San Diego, CA, USA). Statistical analysis of the effects of sampling time and pain group upon the observed lipid concentrations were undertaken using two-way robust Wilcoxon analyses (available with the function raov in the Rfit package version 0.22 of the R computer programme [22, 23]). Correlations between AEA, 2-AG, related NAEs and linoleic acid-derived oxylipins and self rated pain scores were conducted with Spearman’s correlation coefficients (GraphPad Software). For comparison between independent correlation coefficients, the standard Fisher’s transformation was used with an on-line calculator (http://vassarstats.net/index.html, URL checked on 19 February 2016). The false discovery rate (FDR) by Benjamin and Hochberg [24] was used to control for the expected proportion (5 %) of incorrectly rejected null hypotheses (i.e. false discoveries) due to multiple comparisons.

## Results

### Subject characteristics

Subject characteristics are presented in Table 1. There were no significant differences in the distribution of the time of day of sampling (morning vs. afternoon), the body weight, BMI and number of nicotine users among the subjects. A significant group difference for age was seen, due to a difference between the CWP and NP groups (CWP > NP, *P* < 0.05, Dunn’s multiple comparisons post-hoc test). The scores for the average pain during the last week (“NRS_week_”) were greater for the CWP group than the NP group, whereas the current pain scores (“NRS_day_”) were not significantly different between the two groups.

**Table 1:** Subject characteristics. Data for background variables are presented with median and range for all groups. Non-parametric statistics were used since there was an unequal distribution in the number of subjects between the CWP group and the other two groups, and since in many cases (e.g. body weight), the data were not normally distributed. The number of nicotine users is presented as the numbers and percent in group

	Control (*n* = 27)	Localised neck pain (NP, *n* = 35)	Chronic widespread pain (CWP, *n* = 15)	*P*-value
Age [years]	52 (25–61)	49 (26–64)	58 (41–65)	0.029^a^
Height [cm]	166 (158–180)	167 (156–177)	168 (153–177) ^−2^	0.73^a^
Weight [kg]	64 (50–88) ^-1^	64 (51–100) ^−1^	67 (55–110) ^−2^	0.82^a^
BMI [kg x m^2^]	23 (19–31) ^−1^	24 (19–32) ^−1^	24 (21–38) ^−2^	0.59^a^
NRS_day_	-	2 (1–6)	3 (1–8)	0.28^b^
NRS_week_	-	4 (1–8)	6 (2–7)	0.016^b^
Pain duration [months]	-	42 (5–288) ^−1^	222 (120–420) ^−1^	<0.0001^b^
Nicotine users [No.]	4 (15 %)	4 (11 %)	3 (20 %)	0.73^c^
Sampling time^d^	15 M, 12A	17 M, 17A^−1^	5 M, 9A^−1^	0.48^c^

In the table, the superscripts −1 and −2 indicate the number of missing data for the variable and group in question. Thus, for example, information on body weight was only available for 26 controls

*BMI* Body mass index, *NRS* numerical rating scale 0–10. Statistical tests, *p*-value < 0.05 is considered significant

^a^ Kruskal-Wallis test, ^b^ Mann–Whitney-*U* test, ^c^Chi squared test, excluding the cases when sampling time was not known. ^d^Sampling time is shown as morning (M) and afternoon (A)

### Plasma levels of endocannabinoids, related NAEs and linoleic acid-derived oxylipins in controls and subjects with CWP and NP

**Fig. 1:**
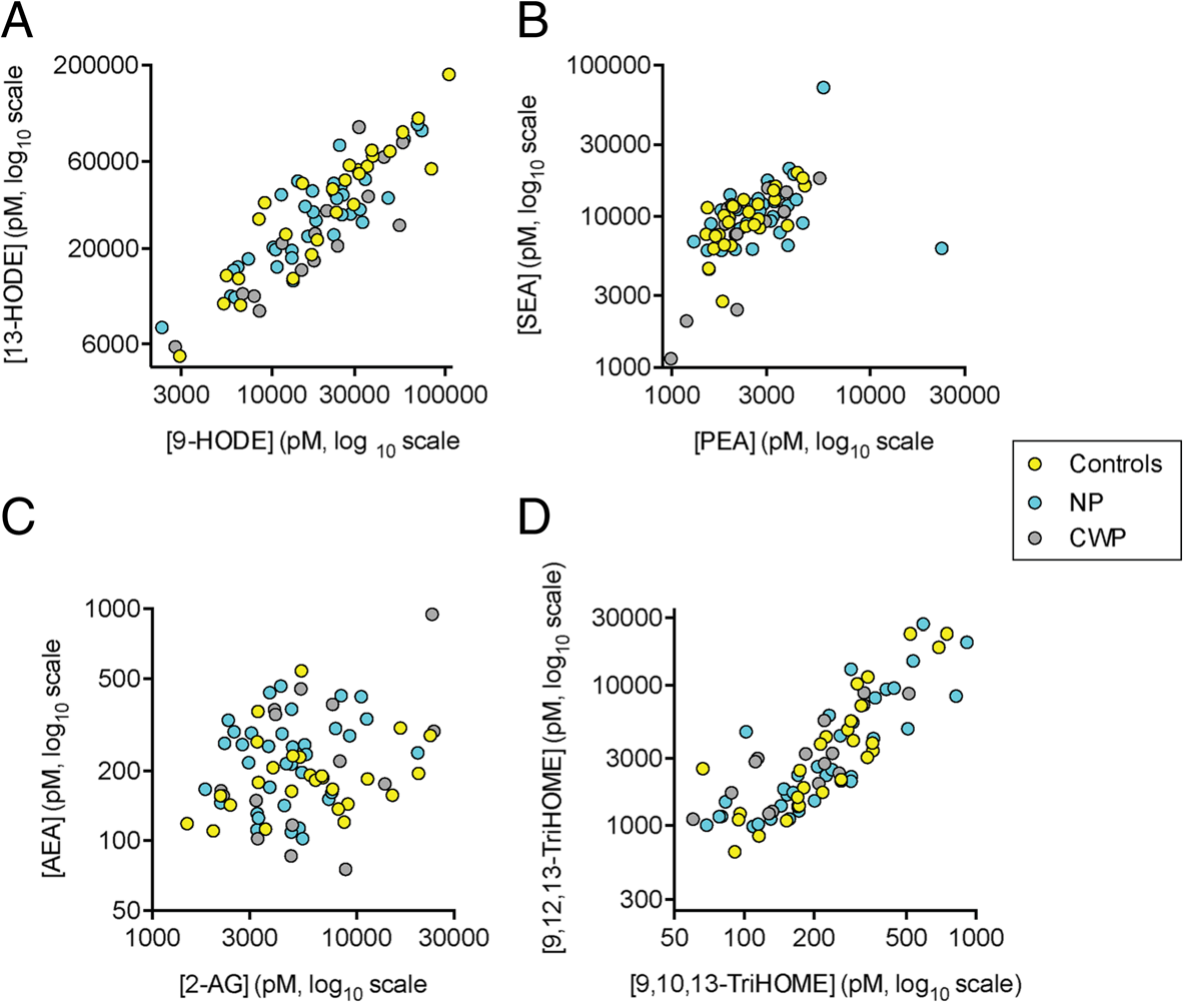
Individual values of **a** 9- and 13-HODE; **b** PEA and SEA; **c** 2-AG and AEA; and **d** 9,10,13-TriHOME and 9,12,13-TriHOME for the controls (*N* = 27), NP (*N* = 35) and CWP (*N* = 15) participants. The Spearman’s rho values for the correlation between the lipid pairs for controls, NP and CWP participants, respectively, were: Panel **a** 0.91, 0.78 and 0.88, all *P* < 0.001); Panel **b** 0.70 [*P* < 0.0001], 0.49 [*P* = 0.0028] and 0.70 [*P* = 0.0048]; Panel **c** 0.23, 0.13 and 0.32, all *P* > 0.2); Panel **d** 0.86 [*P* < 0.0001], 0.85 [*P* < 0.0001] and 0.74 [*P* = 0.0023]

Plasma levels of fifteen lipids (AEA and 2-AG, four related NAEs, and seven linoleic acid-derived oxylipins including 9- and 13-HODE, and two arachidonic acid-derived oxylipins, 5-HETE and 8,9- DiHETrE) were quantified in serum samples from 27 controls, 15 women with CWP and 35 women with NP. The plasma concentrations of 9- and 13-HODE were highly correlated for all three groups (P for Spearman rank correlation coefficients <0.001, Fig. 1a) and a similar pattern was seen for the correlation between the two NAEs PEA and SEA (Fig. 1b). In contrast, the plasma concentrations of the two endocannabinoids were not significantly correlated (Fig. 1c). This is not merely a reflection of potential quantification issues for the low abundant AEA, since 9,10,13-TriHOME, which has a similar abundance to AEA, is very highly correlated to 9,12-13-TriHOME, which has a similar abundance to 2-AG (Fig. 1d).

The median values and interquartile ranges are shown for all fifteen lipids in Table 2, stratified both on the basis of the condition (control, NP, CWP) and of the time of sampling (morning and afternoon). The *P* values for two-way robust Wilcoxon analyses [22, 23] are also given in the Table. In no case did the ANOVA *P* values for the condition as main effect reach significance. Significant interactions were seen for the condition × time of sampling for LEA (*P* = 0.024) and 13-oxo-ODE (*P* = 0.047), but these levels were below the threshold for significance upon implementation of the false discovery rate approach of Benjamini and Hochberg [24]. A similar result was seen when the cases were stratified into two BMI groups: BMI in the normal range (18.5–24.9 kg/m^2^; *N* = 57 in total) and overweight/obese (BMI ≥25 kg/m^2^; *N* = 22 in total; only 6 individuals were obese [BMI ≥30 kg/m^2^] and this was adjudged to be a too small sample size). The main effect of condition again did not reach significance for any of the lipids (*P* > 0.27) and the only significant finding was an interaction condition × BMI for AEA (*P* = 0.027, otherwise *P* > 0.14 for the interactions, two-way robust Wilcoxon analyses).

**Table 2:** Levels of endocannabinoids, linoleic acid-related oxylipins and related lipids in blood samples from controls and subjects with CWP or NP

		Morning sampling		Afternoon sampling		
Lipid	Condition	Median	iqr	Median	iqr	*P* value
2-AG	Control	5382	4342	6386	11471	C: 0.35
	NP	4325	4139	4820	3483	**T: 0.047**
	CWP	3279	2865	8303	14621	C x T: 0.17
AEA	Control	179	63	189	140	C: 0.14
	NP	260	195	217	121	T: 0.50
	CWP	156	215	221	227	C x T: 0.21
PEA	Control	1930	714	2728	1197	C: 0.35
	NP	3153	1531	2726	1263	T: 0.19
	CWP	2084	1510	2961	2266	C x T: 0.074
SEA	Control	8439	5048	11220	5427	C: 0.97
	NP	9060	5435	9982	5219	T: 0.072
	CWP	11310	11256	10690	7528	C x T: 0.56
OEA	Control	926	1685	1767	988	C: 0.20
	NP	1861	1145	1761	1009	T: 0.81
	CWP	1760	1364	1291	1875	C x T: 0.080
LEA	Control	619	184	950	455	C: 0.39
	NP	961	425	780	431	T: 0.32
	CWP	619	806	710	1220	**C x T: 0.024**
9-HODE	Control	13247	31114	26587	20201	C: 0.75
	NP	23724	11755	13251	16879	T: 0.73
	CWP	23943	36239	17527	29853	C x T: 0.11
13-HODE	Control	35679	43458	43935	37629	C: 0.22
	NP	39311	13634	20330	17000	T: 0.49
	CWP	24137	44109	17188	36591	C x T: 0.12
9,10-DiHOME	Control	4498	12601	7585	12910	C: 0.49
	NP	6494	8772	4056	4132	T: 0.39
	CWP	3294	13986	2054	4791	C x T: 0.32
12,13-DiHOME	Control	15725	15768	15935	14365	C: 0.24
	NP	13637	7522	10021	11471	T: 0.57
	CWP	9716	14338	5342	12263	C x T: 0.70
9,10,13-TriHOME	Control	214	189	284	144	C: 0.63
	NP	201	239	289	209	T: 0.36
	CWP	210	171	221	163	C x T: 0.85
9,12,13-TriHOME	Control	2131	3191	3731	4845	C: 0.98
	NP	1657	4282	4173	6797	T: 0.41
	CWP	3007	2834	2761	5927	C x T: 0.51
13-oxo-ODE	Control	1485	1002	1477	1799	C: 0.51
	NP	1781	1513	1146	1498	T: 0.64
	CWP	1188	2179	1280	1423	**C x T: 0.049**
5-HETE	Control	718	437	1065	761	C: 0.93
	NP	907	692	766	538	T: 0.28
	CWP	745	770	852	822	C x T: 0.10
8,9-DiHETrE	Control	145	55	146	45	C: 0.39
	NP	180	74	156	56	T: 0.18
	CWP	164	171	125	123	C x T: 0.77

Data are given as medians and the interquartile ranges (iqr) for the concentrations in pM. The sample sizes for the samples taken during the morning and afternoon, respectively, were: control, 15 and 12; NP, 17 and 17; CWP 5 and 9. *P* values from two-way robust Wilcoxon analyses [22, 23] show main effects of condition (“C”), sampling time (“T”) and the interaction C × T. *P* values <0.05 are shown in bold text

### Association of the plasma levels of endocannabinoids, related NAEs and linoleic acid-derived oxylipins with the NRS pain scores for the CWP and NP patients

The patients were asked to rate their pain on the day of blood sampling (“NRS_day_”) and for the week prior to the blood sampling (“NRS_week_”). The NRS_day_ and NRS_week_ scores (median, with range in brackets) for the NP group were 2 (1–6) and 4 (1–8), respectively. The corresponding scores for the CWP group were 3 (1–8) and 6 (2–7), respectively (Table 1).

**Fig. 2:**
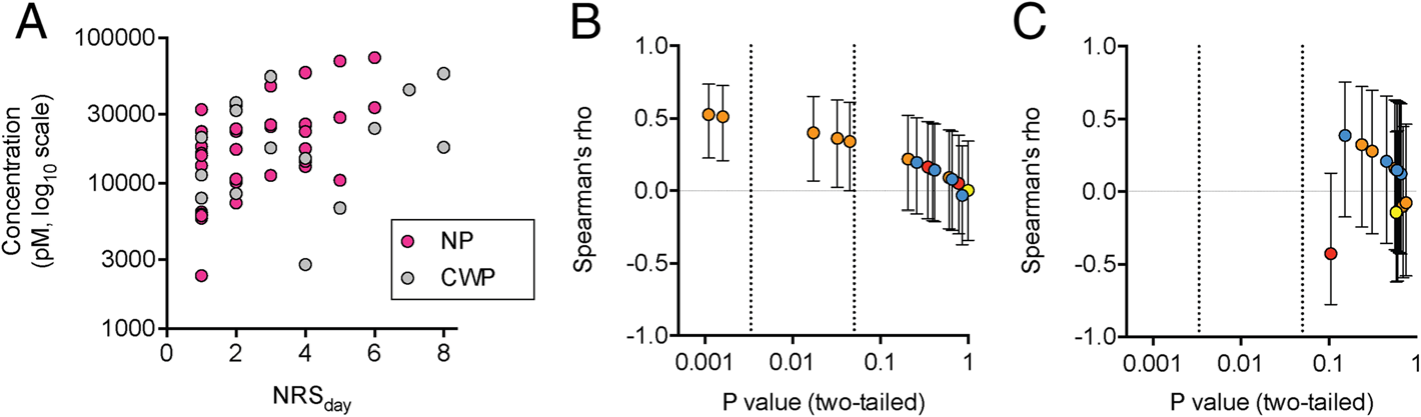
Panel **a** 9-HODE concentrations for the pain group samples plotted against the NRS_day_ scores. Panels **b** and **c** non-parametric correlations between the fifteen lipids studied and the NRS_day_ scores for B, NP and C, CWP participants. The y-axes show the values and 95 % confidence limits for the lipids colour-coded on the basis of their group: orange for linoleic acid-derived oxylipins, red for endocannabinoids, blue for related NAEs and yellow for arachidonic acid-derived oxylipins. The x-axes are the corresponding *P* values for the correlation coefficients, with the vertical lines showing cut-offs at *P* = 0.0033 (the Benjamini and Hochberg [24] 5 % false discovery rate limit for the dataset) and at *P* = 0.05. Note that in Panel **c**, the value for 8,9- DiHETrE (rho 0.14, *P* = 0.41) is hidden behind another data point

Bivariate (zero-order) Spearman rank correlation coefficients were calculated for the correlations between the lipid concentrations and the NRS_day_ scores for the CWP and NP patients. An example of the raw data is shown for 9-HODE in Fig. 2a, and the correlation coefficients are given in Table 3 and presented visually in Fig. 2b and c. The data points are colour coded on the basis of their group (orange for the linoleic acid-derived oxylipins, red for the endocannabinoids, blue for the related NAE’s and yellow for the two arachidonic acid-derived oxylipins). Given that there are multiple comparisons, we have presented the significance levels “as is”, showing two vertical lines: one at *P* = 0.05, and one at *P* = 0.0033, which represents the limit using the false discovery rate approach of Benjamini and Hochberg [24] for the 30 comparisons. For the NP cases, five of the linoleic acid-derived oxylipins (from left to right in Fig. 2b: 13-HODE, 9-HODE, 13-oxo-ODE, 12,13-DiHOME and 9,10-DiHOME) reached significance at the *P* < 0.05 level, and the P values for the two HODEs were smaller than the 5 % Benjamini and Hochberg [24] cut-off value. For the CWP cases, none of the correlations reached significance (Fig. 2b). However, it is important to consider the significance of the difference rather than the difference in the significance [25]. Using the Fisher r-to-z transformation, the significance of the differences for the correlation coefficients for 9- and 13-HODE, 9,10- and 12,13-DiHOME and 13-oxo-ODE were determined. In no case was the difference significant (*P* > 0.13). In consequence, we have also presented the data for the combined NP + CWP groups in Table 3.

**Table 3:** Spearman’s zero- and first-order Spearman rank correlation coefficients between the pain scores and the plasma lipid concentrations

Parameter:	NRS_day_			NRS_day_	NRS_day_	NRS_week_	Pain duration
Pain Group:	NP			CWP	NP + CWP	NP + CWP	NP + CWP
Controlling for:	-	time	BMI	-	-	-	-
N:	35	31	34	15	50	50	48
2-AG	0.05	0.06	0.14	−0.43	−0.10	−0.08	0.07
AEA	0.16	0.13	0.23	0.14	0.11	0.22	−0.05
PEA	−0.03	0.03	−0.01	0.21	0.01	0.12	−0.10
SEA	0.14	0.23	0.18	0.12	0.13	0.05	−0.07
OEA	0.20	0.23	0.16	0.39	0.25	0.16	−0.10
LEA	0.08	0.14	0.05	0.15	0.11	0.23	−0.17
9-HODE	0.51**	0.49**	0.56***	0.28	0.43**	0.06	0.06
13-HODE	0.53**	0.54**	0.54**	0.13	0.35*	−0.01	−0.12
9,10-DiHOME	0.34*	0.33	0.30	−0.13	0.11	0.06	−0.19
12,13-DiHOME	0.36*	0.34	0.33	−0.10	0.13	0.07	−0.16
9,10,13-TriHOME	0.09	0.06	0.05	0.32	0.13	−0.10	−0.08
9,12,13-TriHOME	0.22	0.14	0.20	0.16	0.19	−0.08	0.01
13-oxo-ODE	0.40*	0.38*	0.46**	−0.08	0.23	0.05	−0.15
5-HETE	0.00	−0.06	0.04	−0.14	−0.03	0.02	0.08
8,9-DiHETrE	0.15	0.11	0.18	0.15	0.12	0.09	−0.01

Zero-order correlations are shown where the symbol “-“is given in the row “Controlling for”. First-order correlations controlling for the exact time of day of sampling or for BM were calculated according to the method of Lehmann [33]

***P < 0.001, **P < 0.01, *P < 0.05, otherwise not significant

Table 3 also presents the first-order Spearman rank correlation coefficients for the NP cases taking into account the exact time of sampling (*N* = 31; in the remaining four cases, the exact time of sampling was not available in the dataset) and the BMI (*N* = 34). The correlation coefficients were very similar to the bivariate coefficients between the lipids and the NRS_day_ scores, indicating that the findings described above are retained when controlled for time of sampling or for the BMI of the individuals. Further, first-order Spearman rank correlation coefficients for the NP cases were not changed when controlling for either age (0.52 and 0.56, *N* = 35) or pain duration (0.52 and 0.53, *N* = 34; data for 9- and 13-HODE, respectively).

Spearman rank correlation coefficients were also calculated for the NP, CWP and NP + CWP groups for the correlation between all the lipid concentrations and the NRS_week_ scores and pain duration. In no case was a significant correlation found (see Table 3 for the combined NP + CWP groups).

## Discussion

In the present study, we have utilised a series of blood plasma samples to assess whether differences in endocannabinoids, related NAEs and/or linoleic acid-derived oxylipins are seen in participants with NP or CWP, and whether the blood plasma levels of these lipids are associated with the pain experienced by the participants. The participants were well-characterised with respect both to their current pain and to their ongoing pain, and a strict blood sampling protocol was used whereby blood was rapidly cooled, processed at 4 °C and stored at −80 °C. Limitations of the study are the relatively small sample sizes, given its exploratory nature, and the fact that since the samples were originally taken as part of a different study, the time of sampling was not optimised for an analysis of the lipids investigated here. However, we have controlled for this by including morning/afternoon as a factor in our analyses of the whole group and by undertaking first order correlations between the NRS_day_ scores with the lipid levels controlling for the exact time of sampling for the CWP group. A similar approach was also used to rule out BMI as a potential confounding factor. Nevertheless, effects of other factors, such as differences in food intake, physical activities and other comorbid disease upon the observed plasma levels of the lipids cannot be ruled out.

With respect to the comparisons between controls and the pain groups, no significant changes in lipid levels were seen. The only study, to our knowledge, investigating the current lipids in blood from cases of this type was that of Kaufmann et al. [14], who found a considerably higher AEA concentration in the plasma of 22 individuals with fibromyalgia (17 ♀, 5 ♂, average age 51 years) than for 22 healthy volunteers (17 ♀, 5 ♂, average age 53 years). Although that study used volunteers of both genders, whereas our study was confined to women, the most likely explanation for the difference between their findings and the present study is the choice of the patients. Both studies investigated participants with long-standing disease (>10 years). The individuals in the Kaufmann et al. [14] study scored highly in the fibromyalgia impact questionnaire and had subjective pain scores of 6.4 ± 1.4 (mean ± SEM) as assessed by a visual analogue scale. Our study investigated patients defined as CWP rather than specifically as fibromyalgia, i.e. a potentially more heterogeneous population, and had median NRS_day_ scores of 3.

Perhaps the most interesting result of the present study is the positive association between 9- and 13-HODE levels and the NRS_day_ scores. The fact that both HODEs reached similar significances is not surprising given the high correlation between their plasma values (Fig. 1a). As pointed out earlier, a significant association does not imply causality, and so both NRS_day_ scores → HODE levels and HODE levels → NRS_day_ scores should be considered, as should the possibility that a third factor affects both NRS_day_ scores and HODE levels, thereby inducing a significant correlation between these two variables. A recent report in this Journal presented evidence of ongoing inflammatory processes in individuals with work related neck/shoulder complaints [26]. In healthy individuals and in patients with asthma, a chronic inflammatory disease, provocation by exposure to subway air leads to increased levels of linoleic acid-derived oxylipins in bronchoalveolar lavage samples [27]. Abnormal levels of linoleic acid-derived oxylipins are seen in blood serum from patients with Achilles tendinopathy [15], and an increased rate of oxylipin production from linoleic acid is seen in dental pulp samples from patients with inflammatory dental pain [28]. However, in the present study, plasma HODE levels were not significantly different between groups and in participants with pain. In any case, the source of these lipids is not solely from the affected region of the body, and so the data cannot shed light upon the underlying pathology of the pain conditions.

With respect to HODE levels → NRS_day_ scores, both 9- and 13-HODE are capable of activating TRPV1 receptors on capsaicin-sensitive trigeminal neurons [16] and the use of either antibodies to the two HODE derivatives or a TRPV1 antagonist reduces the allodynia produced by thermal injury to the rat paw [29]. In our view, a more likely explanation of our findings, given that hyperalgesia is a feature of neck pain [30], is that the participants with pain are more sensitive than the healthy controls to the local nociceptive effects of normal levels of circulating HODEs upon TRPV1 receptors, and thereby show the associations that we have found. Certainly, in animal models, persistant nociception triggered by nerve growth factor results in increased nociceptive responses to TRPV1 activation by capsaicin [31], and inflammation of the masseter muscle increases TRPV1 expression in the inflamed muscle, but not in the contralateral muscle [32]. Although this is an attractive (albeit tenuous) hypothesis, it is based on our exploratory data and further investigations, preferably in studies with longitudinal designs, into the link between musculoskeletal pain and circulating oxylipins are clearly necessary.

## Conclusions

The present study has reported an association between the NRS_day_ scores and the plasma levels of two linoleic acid derivatives, 9- and 13-HODE, in individuals with localised musculoskeletal pain. These data motivate further studies into the role(s) of these oxylipins in musculoskeletal pain.

## Abbreviations

12(13)-DiHOME: 12(13)-dihydroxy-9Z-octadecenoic acid
13-HODE: 13-hydroxy-9*Z*,11*E*-octadecadienoic acid
13-oxo-ODE: 13-oxo-9*Z*,11*E*-octadecadienoic acid
2-AG: 2-arachidonoylglycerol
5-HETE: 5-hydroxy-6*E*,8*Z*,11*Z*,14*Z*-eicosatetraenoic acid
8,9- DiHETrE: 8,9-dihydroxy-5*Z*,11*Z*,14*Z*-eicosatrienoic acid
9,10,13-TriHOME: 9,10,13-trihydroxy-11-octadecenoic acid
9,12,13-TriHOME: 9,12,13-trihydroxy-10*E*-octadecenoic acid
9(10)-DiHOME: 9(10)-dihydroxy-12Z-octadecenoic acid
9-HODE: 9-hydroxy-10*E*,12*Z*-octadecadienoic acid
AEA: arachidonoylethanolamide, anandamide
BHT: butylhydroxytoluene
CB: cannabinoid
CUDA: [[(cyclohexylamino)carbonyl]amino]-dodecanoic acid
CWP: chronic widespread pain
ESI: electrospray ionization
LEA: lenoleoylethanolamide
NAE: *N*-acylethanolamine
NDI: Neck Disability Index
NP: chronic neck pain
NRS_day_: current pain on a numeric rating scale
NRS_week_: average pain during the last week on a numerical rating scale
OEA: oleoylethanolamide
PEA: palmitoylethanolamide
SEA: stearoylethanolamide
TRPV1: transient receptor potential vanilloid
UPLC-MS/MS: ultra performance liquid chromatography coupled to tandem mass spectrometry

## References

[1] Murray CJ, Atkinson C, Bhalla K, Birbeck G, Burstein R, Chou D (2013). The state of US health, 1990-2010: burden of diseases, injuries, and risk factors. JAMA..

[2] Wolfe F, Smythe HA, Yunus MB, Bennett RM, Bombardier C, Goldenberg DL (1990). The American College of Rheumatology 1990 criteria for the classification of fibromyalgia. Report of the Multicenter Criteria Committee. Arthritis Rheum.

[3] Wolfe F, Clauw DJ, Fitzcharles MA, Goldenberg DL, Katz RS, Mease P (2010). The American College of Rheumatology preliminary diagnostic criteria for fibromyalgia and measurement of symptom severity. Arthritis Care Res..

[4] Gerdle B, Björk J, Cöster L, Henriksson KG, Henriksson C, Bengtsson A (2008). Prevelance of widespread pain and associations with work status: a population study. BMC Musculoskeletal Dis..

[5] Gerdle B, Larsson B, Forsberg F, Ghafouri N, Karlsson L, Stensson N (2014). Chronic widespread pain. Increased glutamate and lactate concentrations in the trapezius muscle and plasma. Clin J Pain.

[6] Gerdle B, Kristiansen J, Larsson B, Saltin B, Søgaard K, Sjøgaard G (2014). Algogenic substances and metabolic status in work-related Trapezius Myalgia: a multivariate explorative study. BMC Musculoskelet Disord..

[7] Singh Tahim A, Sántha P, Nagy I (2005). Inflammatory mediators convert anandamide into a potent activator of the vanilloid type 1 transient receptor potential receptor in nociceptive primary sensory neurons. Neuroscience..

[8] Guindon J, Hohmann AG (2009). The endocannabinoid system and pain. CNS Neurol Disord Drug Targets..

[9] Fowler CJ (2012). Monoacylglycerol lipase - a target for drug development?. Br J Pharmacol..

[10] LoVerme J, Russo R, La Rana G, Fu J, Farthing J, Mattace-Raso G (2006). Rapid broad-spectrum analgesia through activation of peroxisome proliferator-activated receptor-α. J Pharmacol Exp Ther..

[11] Suardíaz M, Estivill-Torrús G, Goicoechea C, Bilvao A, Rodríguez de Fonseca F. Analgesic properties of oleoylethanolamide (OEA) in visceral and inflammatory pain. Pain. 2007;133:99–110. 10.1016/j.pain.2007.03.008 17449181

[12] Dalle Carbonare M, Del Giudice E, Stecca A, Colavito D, Fabris M, D’Arrigo A (2008). A saturated N-acylethanolamine other than N-palmitoyl ethanolamine with anti-inflammatory properties: a neglected story. J Neuroendocrinol.

[13] Ghafouri N, Ghafouri B, Larsson B, Stensson N, Fowler CJ, Gerdle B (2013). Palmitoylethanolamide and stearoylethanolamide levels in the interstitium of the trapezius muscle of women with chronic widespread pain and chronic neck-shoulder pain correlate with pain intensity and sensitivity. Pain..

[14] Kaufmann I, Schelling G, Eisner C, Richter HP, Krauseneck T, Vogeser M (2008). Anandamide and neutrophil function in patients with fibromyalgia. Psychoneuroendocrinology.

[15] Gouveia-Figueira S, Nording ML, Gaida JE, Forsgren S, Alfredson H, Fowler CJ (2015). Serum levels of oxylipins in Achilles tendinopathy: an exploratory study. PLoS One..

[16] Patwardhan AM, Scotland PE, Akopian AN, Hargreaves KM (2009). Activation of TRPV1 in the spinal cord by oxidized linoleic acid metabolites contributes to inflammatory hyperalgesia. Proc Natl Acad Sci U S A..

[17] Patwardhan AM, Akopian AN, Ruparel NB, Diogenes A, Weintraub ST, Uhlson C (2010). Heat generates oxidized linoleic acid metabolites that activate TRPV1 and produce pain in rodents. J Clin Invest..

[18] Bjorklund M, Djupsjobacka M, Svedmark A, Hager C (2012). Effects of tailored neck-shoulder pain treatment based on a decision model guided by clinical assessments and standardized functional tests. A study protocol of a randomized controlled trial. BMC Musculoskeletal Dis..

[19] Dworkin RH, Turk DC, Farrar JT, Haythornthwaite JA, Jensen MP, Katz NP (2005). Core outcome measures for chronic pain clinical trials: IMMPACT recommendations. Pain..

[20] Ferreira-Valente MA, Pais-Ribeiro JL, Jensen MP (2011). Validity of four pain intensity rating scales. Pain..

[21] Gouveia-Figueira S, Nording ML (2015). Validation of a tandem mass spectrometry method using combined extraction of 42 oxylipins and 15 endocannabinoid-related compounds including prostamides from biological matrices. Prostaglandins Other Lipid Mediat..

[22] Kloke JD, McKean JW (2012). Rfit: Rank-based estimation for linear models. The R Journal.

[23] R Core Team. R: A language and environment for statistical computing. R Foundation for Statistical Computing, Vienna, Austria. ISBN 3-900051-07-0, URL http://www.R-project.org/201

[24] Benjamini Y, Hochberg Y (1995). Controlling the false discovery rate: a practical and powerful approach to multiple testing. J R Statist Soc B..

[25] Nieuwenhuis S, Forstmann BU, Wagenmakers E-J (2011). Erroneous analyses of interactions in neuroscience: a problem of significance. Nat Neurosci..

[26] Matute Wilander A, Kåredal M, Axmon A, Nordander C (2014). Inflammatory biomarkers in serum in subjects with and without work related neck/shoulder complaints. BMC Musculoskeletal Dis..

[27] Lundström SL, Levänen B, Nording M, Klepczynska-Nyström A, Sköld M, Haeggström JZ (2011). Asthmatics exhibit altered oxylipin profiles compared to healthy individuals after subway air exposure. PLoS ONE..

[28] Ruparel S, Hargreaves KM, Eskander M, Rowan S, de Almeida JFA, Roman L (2013). Oxidized linoleic acid metabolite-cytochrome P450 system (OLAM-CYP) is active in biopsy samples from patients with inflammatory dental pain. Pain..

[29] Green DP, Ruparel S, Roman L, Henry MA, Hargreaves KM (2013). Role of endogenous TRPV1 agonists in a postburn pain model of partial-thickness injury. Pain..

[30] Johnston V, Jimmieson NL, Jull G, Souvlis T. Quantitative sensory measures distinguish office workers with varying levels of neck pain and disability. Pain. 2008;137:257-65. 10.1016/j.pain.2007.08.037 17964075

[31] Eskander MA, Ruparel S, Green DP, Chen PB, Por ED, Jeske NA (2015). Persistent nociception triggered by nerve growth factor (NGF) is mediated by TRPV1 and oxidative mechanisms. J Neurosci..

[32] Simonic-Kocijan S, Zhao X, Liu W, Wu Y, Uhac I, Wang KW (2013). TRPV1 channel-mediated bilateral allodynia induced by unilateral masseter muscle inflammation in rats. Mol Pain..

[33] Lehmann R (1977). General derivation of partial and multiple rank correlation coefficients. Biom J..

